# Oxidative Stress Response Kinetics after 60 Minutes at Different (1.4 ATA and 2.5 ATA) Hyperbaric Hyperoxia Exposures

**DOI:** 10.3390/ijms241512361

**Published:** 2023-08-02

**Authors:** Clément Leveque, Simona Mrakic Sposta, Sigrid Theunissen, Peter Germonpré, Kate Lambrechts, Alessandra Vezzoli, Gerardo Bosco, Morgan Lévénez, Pierre Lafère, François Guerrero, Costantino Balestra

**Affiliations:** 1 Environmental, Occupational, Aging (Integrative) Physiology Laboratory, Haute Ecole Bruxelles-Brabant (HE2B), 1160 Brussels, Belgium; cleveque@he2b.be (C.L.); klambrechts@he2b.be (K.L.); plafere@he2b.be (P.L.); 2 Laboratoire ORPHY, Université de Bretagne Occidentale, UFR Sciences et Techniques, 6 Avenue Le Gorgeu, 93837 Brest, France; francois.guerrero@univ-brest.fr; 3 Institute of Clinical Physiology, National Research Council (CNR), 20162 Milan, Italy; simona.mrakicsposta@cnr.it (S.M.S.); alessandra.vezzoli@cnr.it (A.V.); 4 DAN Europe Research Division (Roseto-Brussels), 1160 Brussels, Belgium; pgermonpre@gmail.com; 5 Hyperbaric Centre, Queen Astrid Military Hospital, 1120 Brussels, Belgium; 6 Environmental Physiology & Medicine Lab, Department of Biomedical Sciences, University of Padova, 35131 Padova, Italy; gerardo.bosco@unipd.it; 7 Anatomical Research and Clinical Studies, Vrije Universiteit Brussels (VUB), 1090 Brussels, Belgium; 8 Physical Activity Teaching Unit, Motor Sciences Department, Université Libre de Bruxelles (ULB), 1050 Brussels, Belgium

**Keywords:** oxygen biology, cellular reactions, human, oxygen therapy, human performance, hyperbaric oxygen therapy, oxygen dose

## Abstract

Hyperbaric oxygen therapy (HBOT) is a therapeutical approach based on exposure to pure oxygen in an augmented atmospheric pressure. Although it has been used for years, the exact kinetics of the reactive oxygen species (ROS) between different pressures of hyperbaric oxygen exposure are still not clearly evidenced. In this study, the metabolic responses of hyperbaric hyperoxia exposures for 1 h at 1.4 and 2.5 ATA were investigated. Fourteen healthy non-smoking subjects (2 females and 12 males, age: 37.3 ± 12.7 years old (mean ± SD), height: 176.3 ± 9.9 cm, and weight: 75.8 ± 17.7 kg) volunteered for this study. Blood samples were taken before and at 30 min, 2 h, 24 h, and 48 h after a 1 h hyperbaric hyperoxic exposure. The level of oxidation was evaluated by the rate of ROS production, nitric oxide metabolites (NOx), and the levels of isoprostane. Antioxidant reactions were assessed through measuring superoxide dismutase (SOD), catalase (CAT), cysteinylglycine, and glutathione (GSH). The inflammatory response was measured using interleukine-6, neopterin, and creatinine. A short (60 min) period of mild (1.4 ATA) and high (2.5 ATA) hyperbaric hyperoxia leads to a similar significant increase in the production of ROS and antioxidant reactions. Immunomodulation and inflammatory responses, on the contrary, respond proportionally to the hyperbaric oxygen dose. Further research is warranted on the dose and the inter-dose recovery time to optimize the potential therapeutic benefits of this promising intervention.

## 1. Introduction

Hyperbaric oxygen therapy (HBOT) is a therapeutical approach based on breathing pure oxygen (O_2_) in an augmented atmospheric pressure. According to the Undersea and Hyperbaric Medical Society (UHMS), this pressure may equal or exceed 1.4 atmospheres (ATA). However, all current UHMS-approved indications require that patients breathe near 100% oxygen while enclosed in a chamber pressurized to a minimum of 2 ATA (https://www.uhms.org/resources/hbo-indications.html) (accessed on 30 June 2023).

Depending on the protocol, the duration of a single session varies from 1 to 1.5 h (without considering the time needed to reach the intended pressure, which may take up to 15 min for pressurization and 15 min for depressurization), the repetition of exposures varies from one to three times daily, with 20 to 60 therapeutical doses to be administered depending on the condition [1]. Frequently, this method utilizes pressures between 2 to 3 ATA. Nevertheless, promising results have also been obtained for certain conditions with pressures less than 2 ATA (1.5 ATA) [2,3], and in some studies, even ‘hyperbaric air’ seems to be of interest [4]. While some protocols accept the use of 6 ATA (e.g., for the treatment of gas embolism), little benefit is usually reported from pressures above 3 ATA as this may be associated with a plethora of adverse effects [5].

Although it has been used for years, the exact kinetics of the reactive oxygen species between different levels of hyperbaric oxygen exposure are still not clearly evidenced and, without much scientific evidence, it is common practice to apply HBOT sessions every 24 h [6]. The need for several sessions to reach a relevant effect is likewise commonly accepted; however, the optimal hyperbaric oxygen levels and the time needed between each session to optimize cellular responses—such as Hypoxia inducible factor (HIF-1α) or nuclear factor kappa β (NF-Kβ), erythroid related factor 2 (NRF2), cellular vesicles and microparticles, Caspase 3 [7,8,9,10]—are still debated and stand solely on observational clinical outcomes. Some recent experimental works have been evaluating the effects of different levels of oxygen on oxidative stress under hypoxic [11,12,13,14], normobaric hyperoxic [15,16,17], and hyperbaric hyperoxic [4,6,7,8] conditions. The encouraging, but also challenging, results lead even to question if some oxygen levels formerly considered as ‘HBOT sham’ [18] may be of therapeutic interest [4,7].

The purpose of this study was to investigate oxidative, inflammatory, and enzymatic reactions following exposures for 60 min at two different (mild or high) levels of hyperbaric oxygen, 1.4 ATA and 2.5 ATA.

The lower level (1.4 ATA) was chosen because it approaches the level of hyperoxia that may be reached during some underwater diving exposures but also because the therapeutical use of such lower oxygen pressures is being debated. The higher level of hyperbaric hyperoxia is the common average therapeutical exposure.

## 2. Results

### 2.1. ROS, NOx, and 8-Isoprostane (8-Iso-PGF2α) Levels after One Hour of Oxygen Exposure at 1.4 and 2.5 ATA

**Figure 1:**
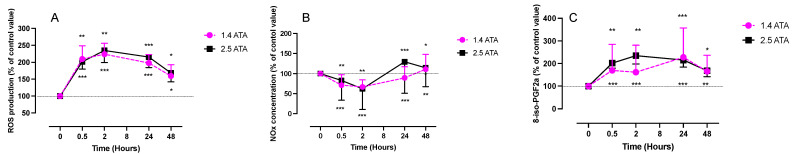
Evolution of ROS (plasma) production rate (**A**), NOx (urine) (**B**), and 8-iso-PGF2a (urine) (**C**) after 60 min of mild (1.4 ATA, n = 6) or high hyperbaric exposure (2.5 ATA, n = 8). Results are expressed as mean ± SD as a percentage of the control value. T0 represents pre-exposure baseline (ROS: 0.18 ± 0.016 μmol·min^−1^) (Nox: 265.7 ± 43.76 μM) (8-iso-PGF2a: 286 ± 124). Intra-group comparisons between results at T0 and each other time point are represented above and below the respective curves. Inter-group comparisons between 1.4 and 2.5 ATA exposure when significant are shown between the 2 curves (*: *p* < 0.05, **: *p* < 0.01, ***: *p* < 0.001).

Both oxygen exposures, mild (1.4 ATA) and high (2.5 ATA), elicited a significant increase of plasmatic ROS production rate with a similar kinetic of rise and decrease. Both oxygen exposures induced a significant increase in ROS production with a peak after 2 h (0.408 ± 0.06 µmol·min^−1^ at 1.4 ATA compared to the baseline, *p* < 0.001; and 0.406 ± 0.06 µmol·min^−1^ at 2.5 ATA, *p* < 0.01). Two-way ANOVA (time post exposure x oxygen level) shows a non-significant difference between the two groups (F (4, 52) = 0.86; *p =* 0.490) (Figure 1A). This peak plateaus for about 2 h, and then the quantity of ROS decreases slowly until 48 h without returning to control levels.

Nitric oxide metabolites (Figure 1B) show changes characterized by a significant decrease; they are the lowest after 2 h for both 1.4 ATA (175.4 ± 45.62 µM, *p* < 0.01) and 2.5 ATA (235.1 ± 121 µM, *p* < 0.001). We observed a reactive increase of nitric oxide metabolites 24 h after 2.5 ATA exposure (496.9 ± 249 µM, *p* < 0.001). Two-way ANOVA (time post exposure x oxygen level) shows a non-significant difference between the two groups (F (4, 52) = 0.855; *p* = 0.497).

For 8-isoprostane (pg/mg creatinine) (Figure 1C), a faster increase after the 2.5 ATA exposure is present compared to the 1.4 ATA exposure. These changes reach a peak plateau 2 h after 2.5 ATA (541 ± 2 02 pg·mg^−1^ creatinine, *p* < 0.01) and around 24 h after 1.4 ATA (560.2 ± 2 09 pg·mg^−1^ creatinine, *p* < 0.001). Two-way ANOVA (time post exposure x oxygen level) shows a non-significant difference between the two groups (F (4, 52) = 0.905; *p* = 0.468).

**Figure 2:**
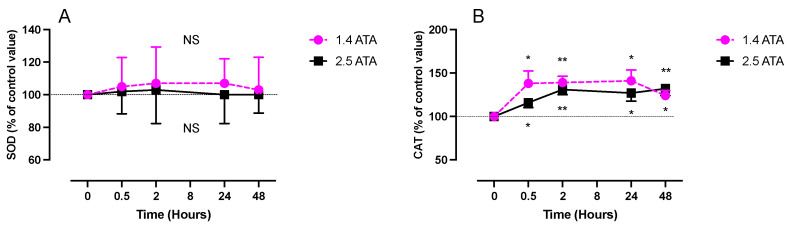
Evolution of SOD (plasma) (**A**) and CAT (**B**), after 60 min of mild (1.4 ATA, n = 6) or high hyperbaric exposure (2.5 ATA, n = 8). Results are expressed as mean ± SD as a percentage of the control value. T0 represents pre-exposure baseline (SOD: 3 ± 1.9 U/mL) (CAT: 24 ± 5.3 U/mL). Intra-group comparisons between results at T0 and each other time point are represented above and below the respective curves. Inter-group comparisons between 1.4 and 2.5 ATA exposure when significant are shown between the 2 curves (ns: not significant; *: *p* < 0.05, **: *p* < 0.01).

Unexpectedly, SOD concentration did not show any change during the 48 h following expositions (Figure 2A). Two-way ANOVA (time post exposure x oxygen level) shows a non-significant difference between the two groups (F (4, 56) = 0.0266; *p* = 0.999), contrary to the CAT levels that increase rapidly after both exposures (1.4 ATA after 30 min: 32.92 ± 11.04 µg/µml, *p* < 0.05; 2.5 ATA after 2 h: 32.62 ± 5.97, *p* < 0.01), whereas two-way ANOVA (time post exposure x oxygen level) shows again a non-significant difference between the two groups (F (4, 52) = 2.47; *p* = 0.056) (Figure 2B). This increase remained constant during the 48 h following both exposures.

### 2.2. Inflammatory Response (IL-6, Neopterin and Creatinine) after One Hour of Oxygen Exposure at 1.4 and 2.5 ATA

**Figure 3:**
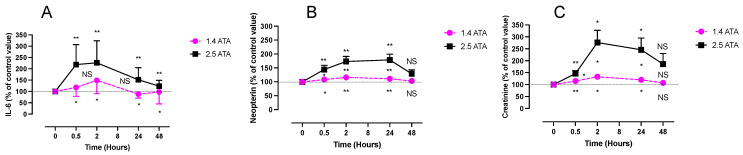
Evolution of the inflammatory response (urine) (IL-6 in (**A**), neopterin in (**B**), and creatinine in (**C**)) after 60 min of mild (1.4 ATA, n = 6) or high hyperbaric exposure (2.5 ATA, n = 8). Results are expressed as mean ± SD as a percentage of the control value. T0 represents pre-exposure baseline (IL-6: 2.818 ± 1.028 pg·mL^−1^; neopterin: 49.62 ± 17.79 mol·mol^−1^; creatinine: 1.1 ± 0.68 g·L^−1^). Intra-group comparisons between results at T0 and each other time point are represented above and below the respective curves. Inter-group comparisons between 1.4 and 2.5 ATA exposure when significant are shown between the 2 curves (ns: not significant; *: *p* < 0.05, **: *p* < 0.01).

Interleukin 6 (IL-6) was measured in plasma samples while neopterin and creatinine were obtained from urine (Figure 3A).

IL-6 shows a significant increase compared to the baseline, presenting its peak at 2 h for the 1.4 ATA group (3.82 ± 0.92 pg/mL; *p* < 0.05) as well as for the 2.5 ATA group (3.36 ± 2.31 pg/mL; *p* < 0.01). Two-way ANOVA (time post exposure x oxygen level) shows a non-significant difference between the two groups at any time (F (4, 48) = 0.390; *p* = 0.815).

Although neopterin rose in both groups, this increase was significantly more pronounced after the 2.5 ATA exposure with a peak at 2 h (83.58 ± 20 µmol/mol creatinine; *p* < 0.01). This is confirmed by the two-way ANOVA (time exposure x oxygen level) of F (4, 56) = 5.32; *p* = 0.001. The plateau was also reached after 2 h in the 1.4 ATA group (57.28 ± 20.8 µmol/mol creatinine; *p* < 0.01). In both groups, values returned to normal after 48 h (Figure 3B).

Similar to neopterin, creatinine (Figure 3C) increased in both groups with a significantly remarkable effect after the 2.5 ATA exposure (2 h post 1.4 ATA: 1.31 ± 0.66 g/L; *p* < 0.01 vs. 2 h at 2.5 ATA: 2.52 ± 0.67 g/L; *p* < 0.01), with a return to the baseline after 48 h. Two-way ANOVA (time post exposure x oxygen level) shows F (4, 56) = 3.43; *p* = 0.014.

**Figure 4:**
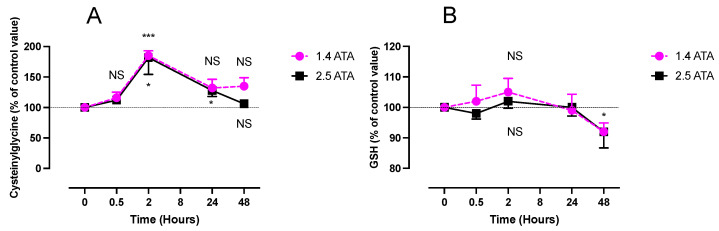
Evolution of cysteinylglycine (plasma) and glutathione (RBC) (GSH) after 60 min of mild (1.4 ATA, n = 6) or high hyperbaric hyperoxia (2.5 ATA, n = 8). Cysteinylglycine (**A**) and glutathione (GSH) (**B**). Results are expressed as mean ± SD. T0 represents baseline pre-exposure values (cysteinylglycine: 38.19 ± 5.715 μmol.L^−1^; GSH: 894.7 ± 176.4 μM). Intra-group comparisons between results at T0 and each other time point are represented above and below the respective curves. Inter-group comparisons between 1.4 ATA and 2.5 ATA of oxygen exposure when significant are shown between the 2 curves (ns: not significant; *: *p* < 0.05, ***: *p* < 0.001).

Cysteinylglycine levels increased rapidly 2 h after 1.4 ATA exposure (11 ± 3.5 µmol·L^−1^, *p* < 0.05) and 2.5 ATA (8.1 ± 3.8 µmol·L^−1^, *p* < 0.001). Values returned to the baseline after 24 h in the 1.4 ATA group and later and after 48 h in the 2.5 ATA group (Figure 4A). We did not observe significant changes in both groups with two-way ANOVA (time post exposure x oxygen level): F (4, 48) = 0.498; *p* = 0.737.

We did not observe any significant changes of glutathione concentrations (GSH), except for a slight decrease 48 h after a 2.5 ATA exposure (918.5 ± 171 µmol·L^−1^, *p* < 0.05), and this is confirmed by two-way ANOVA (time post exposure x oxygen level): F (4, 48) = 0.268; *p* = 0.897 (Figure 4B).

## 3. Discussion

Our study is, to the best of our knowledge, the first to compare the kinetics of a one-hour exposure to mild (1.4 ATA) and high (2.5 ATA) hyperbaric oxygen dose in healthy subjects.

Based on very recent data, we were motivated to explore a new perspective, which involves viewing oxygen not merely as a conventional drug but rather as a powerful trigger for complex molecular reactions [19]. Fundamentally, this process operates through the concepts of ‘dose-response’ and ‘dose-time’, inducing various effects such as oxidative stress, metabolic alterations, and inflammation [20], a phenomenon widely recognized in pathological conditions [21].

Just a decade ago, reactive oxygen species (ROS) were primarily believed to induce harmful effects and were only linked to various pathological conditions [22]. Over time, this perspective shifted, and it was recognized that the presence of reactive oxygen species (ROS) in cells suggests that their production may trigger specific beneficial effects [23]. Our results seem to confirm the reality of such effects at these levels of oxygen breathing.

Firstly, we observed a similar kinetic of plasma reactive oxygen species (ROS) at 1.4 ATA (140 kPa) and 2.5 ATA (250 kPa). The peak production is reached at around 2 h and remains above the baseline level for 48 h. This similar evolution of ROS between the two exposures is an intriguing observation, which suggests that despite the very different oxygen dose, comparable rates of ROS production occur (this is statistically confirmed by two-way ANOVA). Our results apparently contradict a recent study that assessed the effect of HBOT on oxidative markers and immune response [6]. Indeed, in this study, no significant modification of plasma ROS was found following a single HBOT session of 75 min of breathing 100% oxygen through a tight-fitting facemask at a pressure of 240 kPa (2.4 ATA) with two 5 min ‘air-breaks’ (breathing normal air) and a 10 min pressure reduction. Our protocol involved a 60 min exposure at 1.4 ATA and 2.5 ATA with 100% oxygen without any ‘air-breaks’. Although slightly different, our protocol demonstrates a significant increase of ROS production already 30 min after the end of the exposure. While it may be possible that the ‘air-breaks’ that were performed (twice for 5 min) were sufficient to drastically reduce oxidative stress, our results are not able to confirm this hypothesis. More research with a different experimental setup would be needed to assess this difference [24].

Secondly, we observed an increase in creatinine levels; these may be due probably to the vasoconstriction of renal afferent arterioles caused by reactive oxygen species (ROS); this constriction is more pronounced (significant two-way ANOVA) for the higher oxygen exposure (2.5 ATA). The peak of the creatinine increase occurs when nitric oxide metabolite (NOx) levels are at their lowest (in both exposures). This suggests the following mechanism: during hyperoxic exposure, the induced vasoconstriction reduces renal or other organs’ in-flow of blood. This decrease in blood in-flow reduces shear stress and, consequently, the production of nitric oxide (NO). Concomitantly, the bioavailability of NO is reduced by the presence of ROS that exert a scavenging action, given the fact that there are no enzymatic mechanisms for NO inactivation, while its biological life after synthesis in vessels depends on reactions with hemoglobin in the blood, tissue thiols, molecular oxygen, and the superoxide anion. In conditions of extreme hyperoxia, when there is a sharp increase in the production of superoxide anions, existing NO can be neutralized by O_2_^•−^, leading to decreases in its tissue condition, a weakening of its basal vasorelaxing action, and thus to the development of vasoconstriction. Verification of this hypothesis was addressed some years ago by the Demchenko team by analyzing changes in brain blood flow in response to alterations in the balance between NO and O_2_^•−^ in the brain using hyperbaric oxygenation, an intravenous administration of superoxide dismutase, and an inhibition of NO synthase. The vasodilatory effect of superoxide dismutase in hyperbaric oxygenation was not seen in animals given prior doses of the NO synthase inhibitor [25]. These results provide evidence that one mechanism for hyperoxic vasoconstriction in the brain consists of the inactivation of NO by superoxide anions, decreasing its basal vasorelaxing action.

In fact, during the hyperbaric sessions, without physical activity, only vasoconstriction is present, shear stress is reduced, and a laminar flow is favored.

Under fully developed laminar flow conditions, much of the released NO/nitrite remains concentrated in near-wall fluid laminae, in which nitrite levels can accumulate to vasoactive concentrations in downstream resistance vessels. This local synthesis and delivery minimizes the washout (wasting) of endogenous NO [26]. The resulting vasodilation lowers regional vascular resistance and, in turn, increases regional blood flow as well as wall shear stress in the parent branches, which finally creates a positive feedback effect that causes further local arterial NO release [27]. A certain quantity of NO would thus be ‘non-circulating’ during the HBOT session, explaining the NOx reduction measured directly after hyperbaric oxygen exposure.

We may then hypothesize that after the hyperbaric session, the locally present NO will be released in circulation because of the normal ‘everyday-life’ physical activity, and this could explain its rebound increase after 24 h, persisting up to 48 h. Our results confirm a previous study that did not find a systemic increase in NO during hyperbaric oxygen exposures [28].

The increase in reactive oxygen species (ROS) stimulates catalase (CAT) to initiate the breakdown of H_2_O_2_ into H_2_O and O_2_ [26]. In contrast to normobaric hyperoxia [17], our study highlights an increase in CAT production in response to oxidative stress. It is worth noting that CAT exhibits one of the highest turnover rates among known enzymes, with approximately 40,000,000 molecules per second [29,30]. Unlike other peroxidases, CAT does not generate free radicals. Paradoxically, oxidative stress induced by hypoxia appears to be more difficult to cope with than hyperoxia-induced oxidative stress [12].

Generally, as a response, an increased rate of radical production leads to an increment in the levels of antioxidant enzymes; in fact, CAT showed an increased activity very fast after the 1 h HBO exposure. SOD activity apparently remained unchanged at the end of the HBO session and for the next 48 h (no difference for the two-way ANOVA for time post exposure X oxygen level). Interestingly, at the time of the 15th treatment, a significant decrement in SOD and CAT activities (−20% each) was observed compared to the 1st HBO exposure [31]. Other authors found different results showing no variation even after the 20th hyperbaric session [32]. We do not have a definite explanation for this apparent contradiction, but it has been proposed that most likely, the species involved in SOD and CAT inactivation is singlet oxygen and that following several hyperbaric sessions, the protective reactions against this reactive species may vary.

This stress response includes the transcriptional activation of various genes encoding antioxidant and detoxification enzymes as an attempt to dampen the deleterious effects of increased ROS production. Neutrophils could release antioxidant enzymes and particularly CAT into the extracellular space in order to increase the antioxidant effect surrounding the neutrophil. This secretion could be important to avoid oxidative damage in tissues induced by neutrophil ROS generation. Previous studies showed that CAT is colocalized to the specific granules with peroxisomal and lysosomal proteins such as MPO, hydrolases, and peroxidases [33]. Sureda and coworkers [34] observed that the presence of CAT in these granules, in addition to the detected increase in extracellular CAT activity after neutrophil activation, agrees with the possible existence of antioxidant enzymes released from neutrophils.

This can explain the increase of plasmatic CAT, and this is further confirmed by other results showing an activation of the immune system with the significant increase of neopterin and creatinine. It is worth underlining that the two-way ANOVA calculations for the oxygen pressure exposure show a difference in both parameters. This seems to show that oxygen levels have a selective effect on the immune response, which could have been further confirmed if the CAT difference was significant for both exposures; but the two-way ANOVA did not quite reach a level of significance (*p* = 0.056).

If we consider other antioxidant actors, glutathione (GSH) evolution is somehow complex. Following one of its metabolites, cysteinylglycine (CYSGLY), we see a significant increase 2 h after exposure for both oxygen levels (though it is non-significant for oxygen pressure and time post exposure, according to the two-way ANOVA test). This increase is not paralleled by a GSH decrease. We understand this situation as a growing activity of GSH until 2 h post-oxygen. After this peak, a reduction of its metabolite (CYSGLY) is present until 48 h, where basal levels are again reached (both exposures). This decrease of GSH (48 h after exposure) can be interpreted either as a reduction of its production or as an inability of the GSH production to keep up with the ROS still present.

This latter explanation may be the reason behind the mitochondrial dysfunction present (at least) during the first hyperbaric exposures [35]. Indeed, animal studies show a reduction of the mitochondrial membrane rest potential during the first one to five treatments which stimulates the mitochondrial apoptotic pathway and stimulates reactive biogenesis during the following treatments [28,36].

Contrary to our previous results on normobaric hyperoxia, we observed a direct increase in IL-6, suggesting a stimulation of the NFR2/NF-Kβ pathway [37,38]. Additionally, we observe a simultaneous increase in neopterin production, indicating an immunomodulatory role of hyperbaric hyperoxia [39]. Neopterin is generated in response to a γ-interferon-mediated activation of monocytes and macrophages and thus is a direct product of immune system activation [40,41]. Neopterin has been shown to elicit a cytoprotective action of cerebral tissue [42]. This provides an additional argument regarding the effects of hyperbaric hyperoxia exposure on cognitive improvements [43], and remarkably, both exposure levels show a significant increase but with a higher effect for 2.5 ATA.

This fact seems to show a dose–response effect that will encourage further research on the targeted use of oxygen exposures since, for instance, in lower hyperoxia levels (30% and 100% of FiO_2_), no such increase is observed [17], but it is present during moderate hypoxia (15% of FiO_2_) [12]. One other factor to consider is that cellular responses to oxygen are not necessarily directly proportional to the inspired level of oxygen, as there is not a linear relationship between alveolar oxygen pressures and the quantity of molecular oxygen at the cellular (and mitochondrial) level [19]. Factors such as pulmonary gas transfer efficiency, arterial wall thickness and endothelial ‘health’, and capillary-to-cell distance (as with an increased extravascular fluid compartment, e.g., local oedema or lower capillary density in sclerotic tissue) will all play a role in determining how the inspired oxygen pressure ‘translates’ to an increased or decreased cellular presence of molecular oxygen. Even though the observed effects may not directly be extrapolated to non-healthy patients, our observations in young healthy volunteers offer a clue to the dynamic and kinetics of oxygen-mediated cellular reactions. They show that depending on the oxygen level breathed, some effects are similar while others are markedly different; each observed effect clearly has its own ‘dose-dependent’ kinetic.

### Limitations

Strengths:

- This study is, to our knowledge, one of the first to investigate the kinetics of responses to a single short hyperbaric oxygen exposure at 1.4 ATA and 2.5 ATA.
- The measurements were conducted until 48 h post-exposure and putatively open the avenue to new possible applications for hyperbaric oxygen breathing protocols.

Weaknesses:

- The subject numbers are limited, but the sample can be considered as homogenous since all were healthy participants.
- The analysis was not made in the nucleus of the cells but in the plasma, red blood cells, or urine; this could be considered a weakness for some, but it would need a thoroughly different experimental setting.

## 4. Materials and Methods

### 4.1. Experimental Protocol

After written informed consent, 48 healthy non-smoking Caucasian subjects (32 males and 16 females) volunteered for this study. None of them had a history of previous cardiac abnormalities or were under any cardio or vaso-active medication.

All experimental procedures were conducted in accordance with the Declaration of Helsinki [44] and approved by the Ethics Committee approval from the Bio-Ethical Committee for Research and Higher Education, Brussels (N° B200-2020-088).

After medical screening to exclude any latent morbidity, participants were prospectively randomized into 6 groups of 6–8 persons each. These groups were divided into hypoxia (10% and 15% of FiO_2_) [12], normobaric hyperoxia (30% and 100% of FiO_2_) [7], and hyperbaric hyperoxia (1.4 ATA and 2.5 ATA). All participants were asked to refrain from strenuous exercise for 48 h before the tests. No antioxidant nutrients, i.e., dark chocolate, red wine, or green tea were permitted in the 8 h preceding and during the study. The subjects were also asked not to dive 48 h before the experiment and not to fly within 72 h before the experiment.

Fourteen participants completed the mild hyperbaric hyperoxia (1.4 ATA, n = 6) and high hyperbaric hyperoxia (2.5 ATA, n = 8) protocols. Age (1.4 ATA: 36.0 ± 12.3 years old (mean ± SD) vs. 2.5 ATA: 38.3 ± 13.6 years old; *p* = 0.75), height (1.4 ATA: 174.2 ± 13.4 cm vs. 2.5 ATA: 177.9 ± 6.9 cm; *p* = 0.51), and weight (1.4 ATA: 67.7 ± 13.0 kg vs. 2.5 ATA: 81.8 ± 19.0 kg; *p* = 0.14) were collected.

Hyperbaric oxygen (oxygen partial pressure: 1.4 ATA; 1400 hPa, n = 6, and 2.5 bar; 2500 hPa, n = 8) was administered for 1 h in a hyperbaric multiplace chamber (Haux-Starmed 2800, Haux-Life-Support GmbH, Karlsbad-Ittersbach, Germany) of the Military Hospital Brussels, Belgium, by means of a tight-fitting orofacial mask connected to the Haux-Oxymaster demand-valve system. A 60 min time was chosen to reduce the risk of oxygen toxicity.

**Figure 5:**
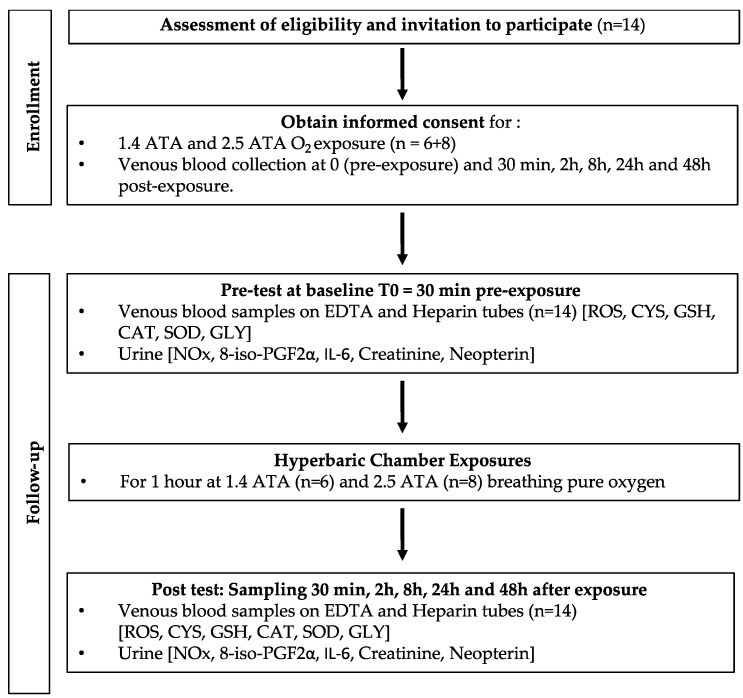
Experimental flowchart.

Blood and urine samples were obtained before exposure (T0) and 30 min, 2 h, 24 h, and 48 h after the end of oxygen administration. The originally planned and accepted protocol included blood sampling 8 h after exposure (see experimental flowchart—Figure 5); for technical reasons, this could not be achieved, but to be consistent with our previous works and also to better depict the sampling duration, we kept this time point in the graphs’ X-axis. Previous experiments have shown that cellular responses after different oxidative exposures may continue for 24 h and even more; we therefore decided to take blood samples up to 48 h [8,10,13,45].

Each blood sample consisted of approximately 15 mL of venous human blood collected in lithium heparin and EDTA tubes (Vacutainer, BD Diagnostic, Becton Dickinson, Italia S.p.A., Milan, Italy). Plasma and red blood cells (RBCs) were separated by centrifugation (Eppendorf Centrifuge 5702R, Darmstadt, Germany) at 1000× *g* at 4 °C for 10 min. The samples of blood cells and plasma were then stored in multiple aliquots at −80 °C until assayed and thawed; an analysis was performed within one month from collection. Urine was collected by voluntary voiding in a sterile container and stored in multiple aliquots at −20 °C until assayed and thawed only before analysis.

### 4.2. Blood Sample Analysis

#### 4.2.1. Determination of ROS by Electron Paramagnetic Resonance (EPR)

An electron paramagnetic resonance instrument (E-Scan—Bruker BioSpin, GmbH, Rheinstetten, Germany) X-band, with a controller temperature at 37 °C interfaced to the spectrometer, was adopted for the ROS production rate, as already performed by some of the authors herein [46,47,48,49]. The EPR measurements are highly reproducible, as previously demonstrated [50]. EPR is the only non-invasive technique suitable for a direct and quantitative measure of ROS. In particular, the spectroscopic technique (EPRS) finds many fields of application, among which is bio-medicine [51]. The reliability and reproducibility of EPR data obtained by the herein adopted micro-invasive EPR method has been already reported previously [52].

Briefly, for ROS detection, 50 µL of plasma were treated with an equal volume of CMH (1-hydroxy-3-methoxycarbonyl-2,2,5,5-tetramethylpyrrolidine), and then 1 mM of the CMH solution was prepared in a buffer (Krebs-Hepes buffer (KHB) containing a 25 μM deferroxamine methane-sulfonate salt (DF) chelating agent and 5 μM sodium diethyldithio-carbamate trihydrate (DETC)) at pH 7.4. The plasma was incubated with CMH (1:1) for 60 s; 50 µL of this solution were placed inside a glass EPR capillary tube in the spectrometer cavity for data acquisition. A stable radical CP (3-Carboxy-2,2,5,5-tetramethyl-1-pyrrolidinyloxy) was used as an external reference to convert ROS determinations in absolute quantitative values (μmol/min). All EPR spectra were collected by adopting the same protocol and obtained by using software standardly supplied by Bruker (Billerica, MA, USA) (version 2.11, WinEPR System).

#### 4.2.2. Superoxide Dismutase (SOD) and Catalase (CAT)

SOD and CAT plasmatic levels were measured by enzyme-linked immunosorbent assay (ELISA kits), according to the manufacturer’s instructions. SOD activity was assessed by Cayman’s SOD assay kit (706002) that utilizes a tetrazolium salt for the detection of superoxide radicals generated by xanthine oxidase and hypoxathine. One unit of SOD is defined as the amount of enzymes needed to exhibit 50% dismutation of the superoxide radical measured in changes in absorbance (450 nm) per minute at 25 °C and at pH 8.0. CAT activity was assessed by Cayman’s assay kit (707002) that utilizes the peroxidic function of CAT. The method is based on the reaction of enzymes with methanol in the presence of an optimal concentration of H_2_O_2_. The formaldehyde produced is measured colorimetrically with Purpald (540 nm) as chromogen. One unit of CAT is defined as the amount of enzymes that will cause the formation of 1 nmol of formaldehyde per minute at 25 °C. All samples and standards were read by a microplate reader spectrophotometer (Infinite M200, Tecan Group Ltd., Männedorf, Switzerland). The determinations were assessed in duplicate, and the inter-assay coefficient of variation was in the range indicated by the manufacturer.

#### 4.2.3. Total Aminothiols (CYS: Cysteine; CYSGLY: Cysteinylglycine and GSH: Glutathione)

Total and reduced aminothiols (CYS: cysteine; CYSGLY: cysteinylglycine; and GSH: glutathione) were measured in erythrocytes (for GSH) and plasma (for CYS and CYSGLY), according to previously validated methods [53,54], at room temperature by an isocratic HPLC analysis on a Discovery C-18 column (250 × 4.6 mm I.D, Supelco, Sigma-Aldrich, St. Louis, MO, USA), eluted with a solution of a 0.1 M acetate buffer, pH 4.0 methanol, 81:19 (*v*/*v*), at a flow rate of 1 mL/min. Fluorescence intensities were measured with an excitation wavelength at 390 nm and an emission wavelength at 510 nm, using a fluorescence spectrophotometer (Jasco, Tokyo, Japan). A standard calibration curve was used for the assayed samples.

### 4.3. Urine Sample Analysis

#### 4.3.1. Nitric Oxide Metabolites (NO_2_ + NO_3_)

NOx (NO_2_ + NO_3_) concentrations were determined in urine via a colorimetric method based on the Griess reaction, using a commercial kit (Cayman Chemical, Ann Arbor, MI, USA), as previously described [55]. Samples were spectrophotometrically read at 545 nm.

#### 4.3.2. 8-Isoprostane (8-Iso-PGF2α)

Levels of 8-iso-PGF2α were measured using an immunoassay EIA kit (Cayman Chemical, Ann Arbor, MI, USA) in urine. This is a biomarker for lipid peroxidation damage assessment. Samples and standards were spectrophotometrically read at 412 nm. Results were normalized by urine creatinine values.

#### 4.3.3. Interleukin-6

IL-6 levels were determined using the ELISA assay kit (ThermoFisher Scientific, Waltham, MA, USA) based on the double-antibody “sandwich” technique, in accordance with the manufacturer’s instructions.

All the above samples and standards were read by a microplate reader spectrophotometer (Infinite M200, Tecan Group Ltd., Männedorf, Switzerland). The determinations were assessed in duplicate, and the inter-assay coefficient of variation was in the range indicated by the manufacturer.

#### 4.3.4. Creatinine and Neopterin Concentrations

Urinary creatinine and neopterin concentrations were measured by the high-pressure liquid chromatography (HPLC) method, as previously described [45], by the Varian instrument (pump 240, autosampler ProStar 410, SpectraLab Scientific Inc., Markham, ON, Canada) coupled to a UV-VIS detector (Shimadzu SPD 10-AV, λ = 240 nm, SpectraLab Scientific Inc. for creatinine; and JASCO FP-1520, λ_ex_ = 355 nm and at λ_em_ = 450 nm, SpectraLab Scientific Inc. for neopterin).

After urine centrifugation at 1500× *g* at 4 °C for 5 min, analytic separations were performed at 50 °C on a 5 µm Discovery C-18 analytical column (250 × 4.6 mm I.D., Supelco, Sigma-Aldrich, Merck Life Science S.r.l., Milano, Italy) at a flow rate of 0.9 mL/min. The calibration curves were linear over the range of 0.125–1 µmol/L and 1.25–10 mmol/L for the neopterin and creatinine levels, respectively. The inter-assay and intra-assay coefficients of variation were <5%.

### 4.4. Statistical Analysis

Statistical analyses were conducted using GraphPad Prism 10 for Mac (La Jolla, CA, USA). Data are given as a percentage of pre-exposure values. Taking the baseline measures as 100%, the percentage or fold changes were calculated for each measurement time, allowing an appreciation of the magnitude of change rather than the absolute values. The difference between the percentage of pre-exposure values and 100% was compared by a two-tailed one-sample *t*-test when normality of the sample was reached, as assessed by the d’Agostino and Pearson tests. Otherwise, the non-parametric Wilcoxon Rank Sum test was used. Comparisons between the 1.4 ATA and 2.5 ATA groups were performed using the unpaired *t*-test (parametric) or Mann–Whitney (non-parametric). In parallel, to assess differences in the baseline values between conditions, a two-way ANOVA was performed with oxygen pressure (1.4 ATA and 2.5 ATA) and time after exposure. When sphericity was not assumed, analysis of variance (ANOVA) main effects and interactions were interpreted with the Greenhouse–Geisser correction. Sidak-corrected multiple comparisons were used to analyze significant interactions and main effects. The alpha level was set at 0.05. Data are presented as mean (M) ± standard deviation (SD). The significance level was set at *p* < 0.05.

The sample size required for a repeated measures analysis of variance was calculated using the G*power calculator 3.1.9.7 software (Heinrich-Heine-Universität, Düsseldorf, Germany) (effect size = 0.65, alpha error = 0.05, Power = 0.80), and the requisite number of participants for this study was 6 in each group, which parallels previous studies [17].

## 5. Conclusions

A short term (60 min) period of mild (1.4 ATA) and high (2.5 ATA) hyperbaric hyperoxia leads to a similar significant increase in the production of reactive oxygen species (ROS), peaking 2 h after exposure and slowly recovering after 48 h, without yet reaching pre-exposure levels at that time.

Similar physiological reactions seem to be present for both very different oxygen doses (1.4 and 2.5 ATA) concerning antioxidant coping strategies. Immunomodulation and inflammatory responses, on the contrary, respond proportionally to the hyperbaric oxygen dose.

Overall, this study provides insights into the cellular effects of a single exposure of hyperbaric hyperoxia, which may either be similar for both doses studied or dose-dependent (such as the immune system response). A further study of the dynamics and kinetics of these effects may lead to new insights as to the optimal dose and administration schedule (inter-dose recovery time) to optimize the therapeutic benefits of intermittent oxygen therapy (either hyperbaric or normobaric). In fact, a more detailed understanding of these effects is paramount to advancing the clinical use of this promising treatment modality.

## Data Availability

Data are available upon request from the authors.

## Abbreviations

8-iso-PGF2a	8-isoprostane
ATA	Atmosphere absolute
CAT	Catalase
CYSGLY	Cysteinylglycine
EPR	Electron Paramagnetic Resonance
FiO_2_	Inspired Fraction of Oxygen
GSH	Glutathione
H_2_O_2_	Hydrogen peroxide
HBOT	Hyperbaric oxygen therapy
IL-6	Interleukine-6
NO	Nitric oxide
NOx	Nitric oxide metabolites
NRF2	Nuclear Factor Erythroid 2 Related—Factor 2
ROS	Reactive Oxygen Species
SOD	Superoxide dismutase
